# Pathway-specific GABAergic inhibition contributes to the gain of resilience against anorexia-like behavior of adolescent female mice

**DOI:** 10.3389/fnbeh.2022.990354

**Published:** 2022-10-13

**Authors:** Chiye Aoki, Adrienne N. Santiago

**Affiliations:** ^1^Center for Neural Science, New York University, New York, NY, United States; ^2^NYU Langone Medical Center, Neuroscience Institute, New York, NY, United States

**Keywords:** electron microscopy, anorexia nervosa, ketamine, hippocampus, prefrontal cortex, exercise, DREADDs, GABRA4

## Abstract

Anorexia nervosa is one of the most debilitating mental illnesses that emerges during adolescence, especially among females. Anorexia nervosa is characterized by severe voluntary food restriction and compulsive exercising, which combine to cause extreme body weight loss. We use activity-based anorexia (ABA), an animal model, to investigate the neurobiological bases of vulnerability to anorexia nervosa. This is a Mini-Review, focused on new ideas that have emerged based on recent findings from the Aoki Lab. Our findings point to the cellular and molecular underpinnings of three ABA phenomena: (1) age-dependence of ABA vulnerability; (2) individual differences in the persistence of ABA vulnerability during adolescence; (3) GABAergic synaptic plasticity in the hippocampus and the prefrontal cortex that contributes to the suppression of the maladaptive anorexia-like behaviors. We also include new data on the contribution to ABA vulnerability by cell type-specific knockdown of a GABA receptor subunit, α4, in dorsal hippocampus. Although the GABA system recurs as a key player in the gain of ABA resilience, the data predict why targeting the GABA system, singularly, may have only limited efficacy in treating anorexia nervosa. This is because boosting the GABAergic system may suppress the maladaptive behavior of over-exercising but could also suppress food consumption. We hypothesize that a sub-anesthetic dose of ketamine may be the magic bullet, since a single injection of this drug to mid-adolescent female mice undergoing ABA induction enhances food consumption and reduces wheel running, thereby reducing body weight loss through plasticity at excitatory synaptic inputs to both excitatory and inhibitory neurons. The same treatment is not as efficacious during late adolescence but multiple dosing of ketamine can suppress ABA vulnerability partially. This caveat underscores the importance of conducting behavioral, synaptic and molecular analyses across multiple time points spanning the developmental stage of adolescence and into adulthood. Since this is a Mini-Review, we recommend additional literature for readers seeking more comprehensive reviews on these subjects.

## Introduction

Anorexia nervosa is characterized by severe voluntary food restriction and body weight loss (American Psychiatric Association [APA], 2013), is often co-morbid with anxiety (Kaye et al., 2009), and with onset that is linked to puberty (Klump, 2013). Nearly 100% of the patients diagnosed with anorexia nervosa exhibit compulsive exercising, which causes extreme body weight loss (Crisp, 1967; Kron et al., 1978; Carrera et al., 2012). Among all mental illnesses, anorexia nervosa ranks the highest in mortality rate, even surpassing that of major depression (Arcelus et al., 2011). The mortality rate among individuals diagnosed with anorexia nervosa is 0.56% per year, 12 times higher than the annual death rate for females 15–24 years old in the general population (Sullivan, 1995). One longitudinal study showed that 16% of patients diagnosed with anorexia nervosa died from causes related to anorexia nervosa within 21 years (Zipfel et al., 2000). Not only the mortality rate but the relapse rate of anorexia nervosa is also unacceptably high, especially when the condition persists into adulthood (Rieux et al., 2002; Walsh, 2013). In spite of the high mortality and relapse rates of this mental illness, there is little agreement on the pharmacotherapy to accompany cognitive-behavioral therapy for halting anorexia nervosa’s maladaptive behaviors (Powers and Bruty, 2009; Aigner et al., 2011; Barbarich-Marsteller et al., 2012). Unfortunately, anxiolytics, such as benzodiazepines, are not efficacious for treating anorexia nervosa (Steinglass et al., 2014). In this Mini-Review, we offer an explanation for the lack of efficacy of benzodiazepines and reasons for hoping that ketamine in combination with cognitive-behavioral therapy could be ameliorative.

While there is little doubt that socio-cultural stressors contribute greatly to anorexia nervosa, use of an animal model enables one to investigate the complementary neurobiological factors yielding individual differences in vulnerability and the gain of resilience to anorexia nervosa (Aoki, 2020) and other eating disorders (Avena, 2020). Such knowledge should help with designing pharmacological treatments that are suitable for adolescents versus adults. Several publications (Aoki et al., 2017; Foldi et al., 2017; Aoki, 2020; Beeler and Burghardt, 2021) provide comprehensive reviews of the mouse model of anorexia nervosa, called activity-based anorexia (ABA) and its contribution to our current understanding of food-restriction and hyperactivity-induced changes in cognitive flexibility and reward circuitry in the general context of corticostriatal circuitry. By contrast, this Mini-Review examines several Aoki lab findings which together present new perspectives regarding (1) Age-dependence of ABA vulnerability; (2) Persistence of ABA vulnerability; (3) Synaptic plasticity of the GABA system that contributes to individual differences in the ability to suppress the maladaptive ABA behavior.

### The emergence of anorexia nervosa among females during adolescence

Approximately 1–4% of the general population of females world-wide are diagnosed with anorexia nervosa at least once in her life-time (Hudson et al., 2007; Keski-Rahkonen et al., 2007; Smink et al., 2014), while this number is 0.2–0.3% for males (Raevuori et al., 2014). The age of onset of anorexia nervosa is most commonly 15–19 (Lucas et al., 1991), especially among females, although the incidence is increasing for girls less than 13 (Nicholls et al., 2011), as is the onset of puberty (Biro et al., 2010). Approximately 25–31% of the patients diagnosed with anorexia nervosa suffer from a chronic and relapsing course (Steinhausen, 2002; Hudson et al., 2007). The danger of relapse is especially high within a year after the first hospitalization (Lay et al., 2002) and among those individuals who are older (Keel et al., 2005; Walsh, 2013; Berends et al., 2018).

### Activity-based anorexia, an animal model of anorexia nervosa

**FIGURE 1:**
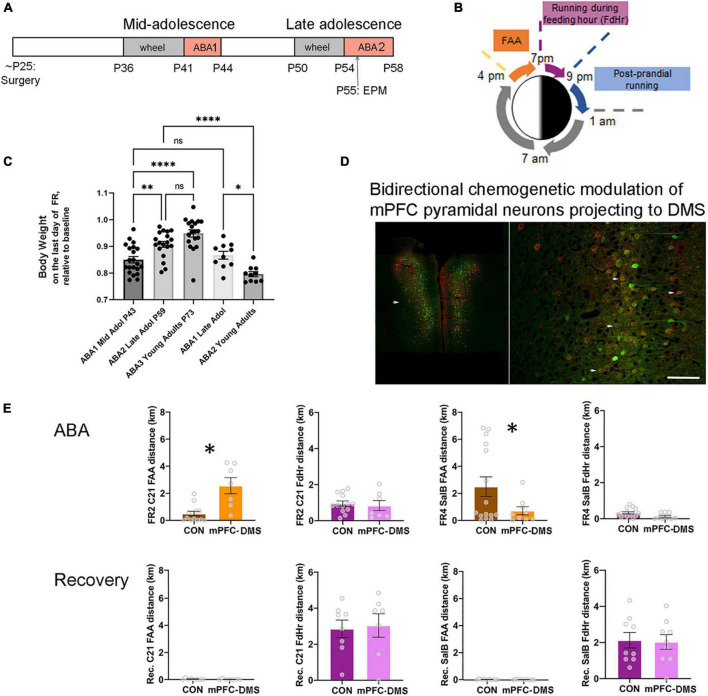
The ABA (activity-based anorexia) paradigm used to reveal the contribution of pathway-specific E/I balance in mPFC to ABA vulnerability during adolescence. **(A)** Timeline. Animals undergo surgery for viral gene transfer to alter the E/I balance at the juvenile stage of ∼P25. From this time, animals are reared in isolation. During mid-adolescence (P36-P44), animals acclimate to the wheel, then undergo 3 days of food restriction (FR) in the presence of a wheel (wheel + FR = ABA1), followed by a period of recovery without a wheel and with *ad libitum* food. In late adolescence (P50-58), animals undergo a second period of wheel acclimation and food restriction (ABA2). Animals are euthanized at the end of ABA2 or after weight restoration from the second ABA, under the condition of wheel access and no food restriction. Wheel running activity is monitored continuously whenever the wheel is in the cage. EPM = elevated plus maze, a test to measure anxiety-like behavior. **(B)** Segments of the 24 h during ABA. ABA begins at 1 pm by removal of food. Food availability becomes restricted to the hours of 7 pm (start of the dark cycle) to 9 pm. FAA develops within a day. **(C)** Comparisons of body weight retention of animals that have undergone their first ABA induction during mid-adolescence (ABA1), followed by two more cycles of ABA induction (ABA2, ABA3) versus those that underwent their first ABA induction during late adolescence, then of ABA2 in adulthood. Each data point represents the body weight datum of one animal. *Indicates *p* = 0.0214; ^**^indicates *p* = 0.0055; ^****^indicates *p* < 0.0001. **(D,E)** Excitability of the cortico-striatal pathway is shown to underlie FAA of animals undergoing ABA2 but not of wheel running under the condition of *ad libitum* food availability during recovery from ABA2. **(D)** The fluorescent images are representative bilateral expression patterns of mCherry, the reporter protein of the excitatory Gq-DREADD and of mCitrine, the reporter protein for the inhibitory DREADD (KORD), by pyramidal neurons in the mPFC. Scale bar = 600 μm (left) and 100 μm (right). **(E)** Each data point in the bar graphs represents the distance run on the wheel during FAA or during the 2 h of food availability (FdHr) of one animal. On the second day of food restriction of ABA2 (FR2) and at the same hour during recovery following ABA2 (Recovery, Rec. bar graphs immediately below the FR2 graphs), C21 was injected intraperitoneally to activate excitatory Gq-M3-DREADDs expressed by mPFC pyramidal neurons projecting to dorsomedial striatum (DMS) (red cell bodies in the fluorescent image). On the fourth day of the second ABA (FR4) and another day during recovery from ABA2 (bar graphs immediately below those of FR4), salvinorin B (salB) was injected to activate the inhibitory DREADDs (KORD) expressed by the overlapping population of pyramidal neurons (Green). Bidirectional chemogenetic modulation of mPFC pyramidal neurons projecting to DMS revealed that activation of these neurons enhances FAA significantly, relative to control animals lacking DREADD expression, while suppression of the activity of these neurons suppresses FAA significantly, compared to the same control animals. The ligands were less efficacious in modulating wheel running during FdHr. Bar graphs represent mean ± SEM. Asterisks indicate *p* < 0.05 by unpaired *t*-test. Fluorescent images are modified from **Figures 4A,B** of Santiago et al. (2021), with permission to reuse from Oxford University Press.

An animal model called activity-based anorexia (ABA) can be used to investigate the neurobiological bases of anorexia nervosa vulnerability. The well-established rat model of ABA (Routtenberg and Kuznesof, 1967; Gutierrez, 2013) has been recapitulated in adolescent female mice (Chowdhury et al., 2013), thereby enabling the use of a wider array of genetically modified models for research. Since the method for inducing ABA in adolescent female mice has been described in detail elsewhere (Aoki, 2020), it will be described only briefly here (Figures 1A,B).

It has long been recognized that rodents acclimated to a running wheel increase their running (exercise) dramatically when food access becomes restricted (Routtenberg and Kuznesof, 1967). Wheel running increases especially during the hours leading up to feeding, called food-anticipatory activity (FAA) (Gallardo et al., 2014) (Figure 1B). FAA is theorized to simulate hunger-evoked foraging (Routtenberg and Kuznesof, 1967; Gutierrez, 2013). Animals can become entrained to as many as four feeding periods per day, exhibiting FAA prior to each feeding period (Luby et al., 2012) but only if the expression of dopamine D1 receptors in dorsal striatum is intact (Gallardo et al., 2014). Alone, food restriction is not life-threatening, because animals can learn to eat more during the limited hours of food availability. However, the combination of food restriction with prior acclimation to a wheel exacerbates running and body weight loss so much more as to cause lethality, unless animals are rescued from the food restriction+exercise environment before losing 20% of their body weight (Aoki et al., 2012; Chowdhury et al., 2013). It is important to note that ABA does not capture all aspects of anorexia nervosa. One of the symptoms of anorexia nervosa that cannot be captured in any animal model is the patient’s concern with the body shape, fear of weight gain and the influence of the “bill board” effects and peer pressure (Berends et al., 2018).

What is most interesting about ABA is that not all adolescent mice succumb to the ABA paradigm: some learn to suppress their excessive running, while others continue with the maladaptive behavior of food restriction-evoked hyperactivity throughout the days of food restriction. Individual differences in ABA vulnerability can be quantified additionally based on body weight loss and reduction of food consumption, as most animals continue to run instead of eat during the limited hours of food availability. The extent of body weight loss correlates with the animal’s running during the 24 h preceding and following body weight measurements, indicating that running is the main cause of body weight loss and that body weight loss propels subsequent running (Chowdhury et al., 2013) due to food restriction-evoked elevation of anxiety (Wable et al., 2015). Although causes of psycho-social origin cannot be captured by ABA, individual differences in hyperactivity and body weight losses can be correlated to synaptic changes within the brain, leading the way for further studies that test causality between molecules within specific brain pathways and persistence or suppression of ABA’s maladaptive behaviors. To pursue synaptic plasticity that underlies individual differences in the gain of resilience against ABA, a bout of ABA can be repeated, after body weight has been restored by *ad libitum* food and removal of the wheel from the environment (Figure 1A).

### Age-dependence of activity-based anorexia vulnerability

In 2013, we reported that approximately 80% of mid-adolescent female mice [postnatal day (P) 41-44] exhibit ABA vulnerability, with this frequency dropping to 50%, when exposed to the combination of food restriction+exercise for a second time (ABA2) in late adolescence (P55-59) (Chowdhury et al., 2013). An update 9 years later with a larger cohort of animals (*N* = 34, instead of 10) indicates rather good agreement with these values: approximately 71% of mid-adolescent female mice exhibited ABA vulnerability, quantified as increases in wheel running per day by more than 10%, relative to the days pre-dating food restriction. As was observed first in 2013, the variance of wheel running increase is large, ranging from 0 to 165%, even within single litters. Mice without the experience of food restriction are dormant during the daylight hours (1 pm – 7 pm, with the dark cycle beginning at 7 pm), but 88% of the mice undergoing ABA induction more than double their FAA running. ABA results in 15% body weight loss by the third day of food restriction (Figure 1C). When exposed to ABA induction for the second time in late adolescence (ABA2, P55-59, *N* = 25), only 50% of food restricted mice increase their wheel running per day. Although 88% of them still exhibit doubling of FAA, body weight loss is much less grave, averaging 9%, instead of 15% during ABA1, even after extending food restriction by another day (*N* = 19) (Figure 1C). This is because animals undergoing ABA2 have learned to eat more efficiently during the limited hours of food access. By the time animals undergo ABA induction for the third time in adulthood (ABA3, *N* = 19), 87.5% of the animals learn to retain 100% of their body weight and the group mean average body weight loss is 5% (Figure 1C), even though 80% of them still exhibit FAA.

A previous report indicated that food restriction+exercise does not evoke ABA upon adult females (4-6 mo postnatal) (Gelegen et al., 2007). Our observations of female ABA mice nearing adulthood (P51-59), corresponding to the ages that mid-adolescent animals undergo ABA2 lead us to a different conclusion. Our assessment is that females in late adolescence are still vulnerable, with 80% of the population exhibiting food restriction-evoked increase in wheel activity by more than 10% and with 70% of them exhibiting more than 100% increase in FAA and losing 13% of their body weight by the third day of food restriction (*N* = 10) (Figure 1C). ABA1 that is delayed to late adolescence does not promote resilience for ABA2 in adulthood (P68-72), since 87.5% increased their daily wheel running and FAA. Moreover, their body weights dropped by 20% during the third day of food restriction, requiring that the food restriction schedule be aborted one day earlier than those undergoing ABA2 in late adolescence (Figure 1C). Thus, compared to the animals that undergo ABA1 during mid-adolescence, those that undergo ABA1 in late adolescence exhibit significantly more severe and prolonged ABA2 vulnerability, due to inadequate feeding relative to energy expenditure caused by food restriction-evoked hyperactivity. This relationship between maturation and severity of ABA2 mirrors the human condition of the increased relapse rate among older individuals with anorexia nervosa (Rieux et al., 2002; Walsh, 2013).

The experience- and age-dependent gain of resilience of animals that undergo ABA in mid-adolescence may be explained, in part, by the fact that adolescent brains are still developing and rewiring (Gerhard et al., 2021; Anderson, 2022). Discussed below are data gathered from the Aoki Lab indicative of contributions made by the still-developing hippocampus and medial prefrontal cortex (mPFC), as they relate to the gain of ABA resilience.

#### Maturation of the hippocampus as it relates to age-dependent gain of resilience to activity-based anorexia during adolescence

In the hippocampus, neurogenesis is more robust during adolescence than in adulthood and the survival of neurons is strongly enhanced by mental and physical skill training during adolescence (DiFeo and Shors, 2017). Dendrites of CA1 pyramidal neurons of female rats undergo a two-fold expansion, followed by retraction *during* adolescence (Chowdhury et al., 2014b). Complexity of the dendritic arbor is influenced in a lamina-specific manner by adolescent social environment (Chen et al., 2018c), food restriction (Chowdhury et al., 2014a), wheel access (Chowdhury et al., 2014a) and by the combined food restriction+exercise - i.e., the ABA-inducing environment (Chowdhury et al., 2014a,b). Contrary to expectation, the severe body weight loss caused by ABA does not retard the development of dendritic complexity. Rather, dendritic complexity is transiently enhanced by ABA in the caudal-ventral CA1, compared to those of controls without food restriction or wheel access, although reduced in the anterior-dorsal CA1 (Chowdhury et al., 2014a). Surprisingly, ABA and food-restricted animals perform better than control animals (no exercise, no food restriction) in the hippocampus-dependent cognitive test of active place-avoidance, once animals have restored their body weight, while animals with *ad libitum* food and wheel access during adolescence exhibit only transient cognitive improvement (Chowdhury et al., 2021). Together, these data indicate that the hippocampus undergoes robust structural remodeling *during* adolescence, providing the cellular and molecular substrates for experience to mold hippocampus-dependent cognitive function.

Although knowledge regarding structural changes of hippocampus is scarce for adolescent patients diagnosed with anorexia nervosa, structural MRI results of the adult population with anorexia nervosa diagnosis (average age 25, *N* = 20) indicate enlargement of the hippocampus among those exhibiting hyperactivity (Beadle et al., 2015). Longitudinal analysis also revealed that the hippocampus reduced in volume after body weight restoration and an accompanying reduction of hyperactivity (Beadle et al., 2015). As for cognitive function, full scale intelligence quotient of children and adolescents diagnosed with anorexia nervosa (ages 11 – 18) is no different from that of age-matched controls, although individuals with anorexia nervosa exhibit significantly worse performance in non-verbal functions and in verbal memory (Kjaersdam Telleus et al., 2015). Notably, impairments in all cognitive functions, including immediate recall, velocity and visual-spatial functions, which are significant during the phase of being underweight, disappear after weight restoration (Lozano-Serra et al., 2014), although those with persistence of amenorrhea at follow up (8 among 22) performed worse on Block Design, delayed recall of visual reproduction and Stroop test than patients with resumed menstruation and control group. These results are unlike the response to refeeding of adults, which shows no improvements or deterioration with time, when associated with more years of malnutrition (reviewed in Lozano-Serra et al., 2014 and Kjaersdam Telleus et al., 2015). A more comprehensive review of hormonal and genetic risk factors during puberty can be found in a previous publication (Klump, 2013).

#### Immaturity of the prefrontal cortex and its connectivity to subcortical targets as it relates to age-dependent vulnerability to activity-based anorexia during adolescence

Prefrontal cortex (PFC) is another brain region still undergoing construction during adolescence. PFC itself and its connectivity to striatum supports self-regulation, habit formation and reward-based learning (Berner and Marsh, 2014). Amygdala is another of the subcortical target regions of the PFC and is well-recognized as the site where fear memory forms and anxiety disorder can develop when connectivity is disturbed (LeDoux, 2000).

Data suggest that striatum peaks in volume prior to puberty onset, and gradually decreases through adolescence and adulthood (Lenroot and Giedd, 2006). Amygdala, like striatum, also attains near-maturation prior to puberty onset (Gogolla et al., 2009; Reh et al., 2020). The early (juvenile stage) maturation of amygdala enables cue-associated fear memory to be more indelible thereafter. In contrast, cortex reaches maturation later than subcortical structures (Reh et al., 2020). Furthermore, among cortical regions, myelination, presumably of afferents and efferents from the PFC to subcortical regions lag behind myelination of cortical regions supporting sensation and movement, thereby remaining incomplete until the end of adolescence (Giedd et al., 1999). Pyramidal neurons of the PFC also undergo spine pruning during adolescence (Juraska and Willing, 2017; Delevich et al., 2021).

The asynchronous and delayed maturation of PFC, relative to the subcortical regions to which it projects, has been proposed to underlie the immaturity of inhibitory control, risky behavior, exaggerated emotional responses (Casey and Jones, 2010; Berner and Marsh, 2014), and the enhanced vulnerability of adolescents to mental illnesses, including eating disorders (Berner and Marsh, 2014). For example, Obsessive-Compulsive Disorder has been linked to weak cortico-striatal connectivity (Bernstein et al., 2016), but also abnormally small striatum and hyperactivity of the cortico-striatal pathway during Obsessive-Compulsive Disorder symptomatic state (Rauch et al., 1997). Healthy adults increase their goal-directed behavior for higher stakes more than for lower-stakes, while healthy adolescents express goal-directed behavior equally for high and low stakes. This difference across the ages has been linked to the developmental changes in the cortico-striatal connection (Insel et al., 2017). The increased vulnerability of adolescent females to anorexia nervosa may be linked to differences in the developmental trajectory of the cortico-striatal pathway. Although the prefrontal cognitive function of set-shifting is comparable for the adolescent patient group diagnosed with anorexia nervosa, relative to healthy controls (Kjaersdam Telleus et al., 2015), maladaptive food choice in anorexia nervosa is linked to stronger PFC-striatum connectivity (Foerde et al., 2015) and lower Stroop test performance during the phase of being underweight (Lozano-Serra et al., 2014). Adolescent females (9-16) but not males, exhibit hyperactivation of PFC during response inhibition (Ordaz et al., 2013). Further details of the development of the cortico-striatal pathway and their relation to bulimia is available in an excellent review by Berner and Marsh (2014).

Within the context of ABA, electron microscopic analysis of GABAergic axon terminals innervating cell bodies of layer 5 pyramidal neurons in the mPFC revealed a 40% enlargement following ABA induction, relative to age-matched controls. Moreover, individuals with the greatest enlargement of GABAergic terminals exhibited the greatest resilience to ABA induction, quantified by their suppression of food restriction-evoked running (Pearson correlation R =−0.8; *p* = 0.009) (Chen et al., 2016).

Most adolescents diagnosed with anorexia nervosa have had childhood diagnosis of anxiety (Kaye et al., 2004), indicating that much can be learned about individual differences in ABA vulnerability from analysis of ABA animals’ subcortical regions. One study indicates that GABAergic plasticity of the amygdala could contribute toward exacerbation of ABA vulnerability. This idea is based on the observation that the occurrence of GABA receptor subtypes containing α4-subunits (to be described in greater detail below) on GABA-interneurons (GABA-INs) increases in the basolateral nucleus of amygdala of animals that have experienced ABA (Wable et al., 2014). Such a change would decrease excitability of GABA-INs, thereby increasing excitability of the glutamatergic projecting neurons and the expression of emotional response to threat-associated sensory cues. Fitting with this prediction, animals that have undergone ABA during adolescence exhibit increased anxiety-like behavior in adulthood (Kinzig and Hargrave, 2010).

These findings from the hippocampus and PFC of animals (1) describe the impact of environmental factors during adolescence, whether it be scarcity-induced stress (e.g., food restriction, social isolation) or enrichment (e.g., wheel) that structurally alter the brain in a region-specific manner; (2) reveal that the structural changes accompany alterations in mood, cognition and ABA vulnerability; and (3) are reminders that structural and functional changes during adolescence can be missed if not probed at multiple time points during adolescence, because the changes can be transient.

### Persistence of activity-based anorexia vulnerability through late adolescence is associated with excessive excitatory outflow of pyramidal neurons forming the pathway originating in medial prefrontal cortex and projecting to dorsomedial striatum

Prompted by an earlier finding that maladaptive food choice in anorexia nervosa is linked to stronger PFC-striatum connectivity (Foerde et al., 2015) and by our findings described above regarding GABAergic innervation of pyramidal neurons in the mPFC of animals that have undergone ABA during adolescence, we focused our analyses on individual differences in mPFC synaptic circuitry and of the excitatory outflow to dorsal medial striatum (mPFC→DMS). We employed the method of multiplexed chemogenetic interrogation, whereby one ligand could be employed to activate the stimulatory Gq-M3-DREADD and another ligand to activate the inhibitory DREADD (KORD) in the same animal (Vardy et al., 2015), following dual viral transduction into mPFC pyramidal neurons with axonal projections to DMS during the juvenile stage, 18 days prior to ABA1 (Figures 1A,D). Chemogenetic activation of the mPFC→DMS pathway of adolescent female mice during ABA2 exacerbates running specifically during FAA. Conversely, chemogenetic suppression of the same pathway of the same animals (on another day of food restriction) suppresses FAA (Figure 1E) (Santiago et al., 2021). The behavioral modulation elicited by the administration of DREADD ligands is specific to the days of food restriction. Once their body weights have been restored by *ad libitum* food in the recovery phase, administration of DREADD ligands has no effect on wheel running (Santiago et al., 2021). The outcome from this control experiment indicates that the chemogenetic modulation of mPFC is far more complex than modulation of a simple motor output pathway for wheel running. The window for modulation is opened by some aspect of ABA, potentially the anxiety induced by food restriction. This supports the idea that the mPFC→DMS excitatory outflow underlies the FAA aspect of ABA vulnerability that persists from mid- to late adolescence, while cellular mechanisms that can dampen the mPFC→DMS outflow may contribute toward the gain of resilience.

### Synaptic plasticity of the GABAergic system in medial prefrontal cortex and hippocampus contributes to individual differences in the ability to suppress the maladaptive behaviors of over-exercising and suppressed feeding

#### mPFC→DMS pathway

**FIGURE 2:**
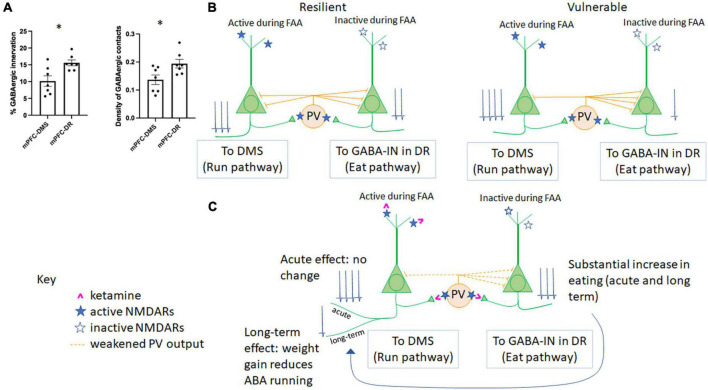
Microcircuitry of the mPFC determines resilience vs. vulnerability to ABA and responsiveness to ketamine. **(A)** GABAergic innervation in the mPFC differs across populations of Layer 5 pyramidal neurons. The percent of somatic pyramidal cell membrane contacted by GABAergic terminals (left) and the density of GABAergic terminals contacting somatic pyramidal cell membrane (right) have been previously reported for pyramidal cells in Layer 5 forming mPFC-DMS (Santiago et al., 2021) and mPFC-DR (Du et al., 2022) subpopulations. Here, we report significantly less GABAergic innervation of the pyramidal cells forming the mPFC→DMS pathway vs. those forming the mPFC→DR pathway (*p* = 0.0104, *t* = 3.031, df = 12 comparing the % membrane innervation; *p* = 0.0271, *t* = 2.515, df = 12, comparing GABA contact density). **(B)** The relationship between GABAergic innervation and individual differences in ABA vulnerability. In the same two publications as those summarized in panel **(A)**, we reported that greater GABAergic innervation of pyramidal cells forming the mPFC→DMS pathway correlates with suppression of running (resilience) while greater GABAergic innervation of pyramidal cells forming the mPFC→DR pathway correlates with reduced feeding (vulnerability). **(C)** Hypothesized mechanism of action of ketamine in the mPFC of animals undergoing ABA. During FAA, pyramidal cells forming the mPFC→DMS pathway are active to produce hyperactive running, while pyramidal neurons forming the mPFC→DR pathway are less active as there is no ability to feed during this time. PV cells are more likely to be active in awake, hyperactively running mice than those mice with suppressed wheel running. Because ketamine (pink) binds to active NMDARs (filled blue star) rather than inactive NMDARs (open blue star), we would expect ketamine that is injected during FAA, 1 h prior to feeding, to weaken firing of PV cells, yielding disinhibition and of the pyramidal neurons of the mPFC→DMS pathway, specifically. Because PV cells are more likely to target mPFC-DR cells vs. mPFC-DMS cells [as shown in panel **(A)**], we would expect the acute response to ketamine to be an increase in mPFC→DR cell firing, resulting in increased feeding, with no net acute effect on pyramidal cells of the mPFC→DMS pathway, resulting in no net acute change in running behavior, which is what we observe during ABA1. Ketamine is known to have sustained effects on behavior. We observed that the increase in feeding is sustained to ABA2, and we also observe reduced running by ABA2, >14 days later (Chen et al., 2018b). This parallels findings in patients with anorexia nervosa, which is that increased feeding helps to reduce disorganized hyperactivity (Crisp, 1967; Kron et al., 1978).

The technique of immunolabeling mPFC tissue dually for GABAergic interneurons (GABA-IN) and DREADD-expressing pyramidal neurons allows for quantitative analysis of axo-somatic inhibitory synapses formed on cell bodies of a subpopulation of Layer 5 pyramidal neurons with axonal projections to DMS. Such quantitative pathway-specific analysis revealed individual differences in the extent of GABAergic innervation of the pyramidal neurons forming the mPFC→DMS pathway (5% to 15%). Animals also varied in the extent to which they lost body weight per day (13% to 19%). The individual differences of the two measurements correlated negatively and significantly (*p* = 0.025; *R* =−.815, *N* = 7) specifically on the day that the mPFC→DMS pathway was chemogenetically activated. This finding is consistent with the idea that recruitment of GABA-IN by pyramidal neurons forming the mPFC→DMS pathway led to stronger feedback inhibition, which in turn contributed to the minimization of ABA vulnerability measured as reduction of body weight loss (Figure 2B).

#### Pathway originating in medial prefrontal cortex and projecting to dorsal raphe

In another set of animals, we tested the influence of chemogenetically modulating the pyramidal neurons in the mPFC projecting to dorsal raphe (mPFC→DR), a pathway known to regulate feeding but never before studied within the context of ABA. Optogenetic stimulation of GABAergic axons in DR which receive direct input from axons originating in the mPFC (Jankowski and Sesack, 2004) promotes feeding through inhibition of the serotonergic neurons in non-ABA animals (Nectow et al., 2017). Contrary to our expectation, chemogenetic stimulation of the mPFC→DR pathway did not appear to promote feeding of ABA animals any more than of control ABA animals without DREADD transduction (Du et al., 2022). The absence of difference between DREADD-transduced and control groups was due to the large variance: the proportion of pyramidal neurons in the mPFC forming the mPFCDR pathway that were activated by the DREADD ligand correlated positively with food intake (*p* = 0.0413, *R* = 0.772). Moreover, correlation analysis of EM data revealed that those animals with greater amount of GABAergic axo-somatic inhibitory synapses onto the DR-projecting mPFC pyramidal cells ate less (*p* = 0.0021; *R* =−0.934). This last negative correlation indicates that individuals with the retraction of GABAergic innervation at somata of pyramidal neurons forming the mPFCDR pathway were the ones that minimized ABA vulnerability by enhancing food intake (Figure 2B).

Importantly, axo-somatic GABAergic innervation of mPFC→DMS cells is significantly less than that of mPFC→DR cells [Figure 2A, modified from data presented in (Santiago et al., 2021; Du et al., 2022)]. Both the percent of somatic membrane contacted by GABAergic terminals (*p* = 0.010, unpaired *T* test) and the density of GABAergic cell contacts (*p* = 0.027, unpaired *T*-test) was less for mPFC→DMS cells vs. mPFC→DR cells (Figure 2A). This unbalanced effect of GABAergic innervation helps to explain the differential in GABAergic control of these two pyramidal cell populations. Overall, these data indicate that plasticity of the mPFC works oppositely for the pyramidal neurons forming the mPFC→DMS pathway and pyramidal neurons forming the mPFC→DR pathway, both of which are under the control of GABA-IN that are recruited by the two populations of pyramidal neurons to mediate feedback inhibition. Specifically, the gain of resilience can involve retraction of the axo-somatic synapses formed by GABA-IN onto DR-projecting pyramidal neurons, leading to enhanced feeding (Figure 2B). The gain of resilience could alternatively or in addition result from expansion of axo-somatic synapses formed by GABA-IN onto DMS-projecting pyramidal neurons, leading to reduced wheel running (Figure 2B). Are there distinct populations of GABA-IN innervating the DR-projecting versus DMS-projecting pyramidal neurons which enable axo-somatic synaptic plasticity to occur in opposite directions? Do pyramidal neurons with different projections “compete” for GABAergic inhibition, so that increased innervation by a GABA-IN onto one population of pyramidal neurons leads to suppressed innervation of the other population of pyramidal neurons? Might there be a third player in the circuit that dictates the balance of GABAergic synaptic expansion/retraction across the two distinct corticofugal pathways? Our results do not yet have answers to these new questions.

#### Dorsal CA1 field of hippocampus

Dorsal hippocampus is an essential brain structure for spatial navigation/exploration, so much so that lesion or pharmacological blockade of synaptic transmission in the dorsal hippocampus impairs active place avoidance (Cimadevilla et al., 2001; Wesierska et al., 2005) and Morris Water Maze performance (Morris et al., 1982). Dorsal hippocampus is also an important structure for anxiety regulation. This latter view is based on the observation that local application of GABA receptor (GABAR) ligands benzodiazepines or the β-carboline inverse agonist into the dorsal hippocamps leads to anxiolytic and anxiogenic effects, respectively (Huttunen and Myers, 1986; Kataoka et al., 1991; Talaenko, 1993).

It has been hypothesized that hippocampal NMDA receptors (NMDARs) serve as “part of a comparator system to detect and resolve conflicts arising when two competing, behavioral response options are evoked concurrently” (Taylor et al., 2014). Although this point of view was based on analyses of choices made by mice faced with competing cues for reward, the two competing behavioral response options for hungry animals could be the drive to explore and forage versus the drive to stay safe, due to stress-induced anxiety. α4 and δ subunit-containing GABA_*A*_Rs (α4βδ-GABA_*A*_Rs) play a unique role in the excitability of CA1 pyramidal neurons of female rodents during adolescence, critically dampening NMDAR-dependent synaptic plasticity at excitatory synapses of pyramidal neurons and active place avoidance performance (Shen et al., 2010) while also increasing anxiety (Shen et al., 2007). The mode of action involving α4βδ-GABA_*A*_Rs in dorsal hippocampus differs from the consistently inhibitory tone mediated by α4βδ-GABA_*A*_Rs in dentate gyrus or the consistently phasic inhibitory action generated by the more widely distributed α1βγ2-GABA_*A*_Rs (Smith and Woolley, 2004). α4βδ-GABA_*A*_Rs are up-regulated at excitatory synapses of pyramidal neurons by ABA in the dorsal CA1, much more than by the experience of exercise, alone or by food restriction, alone (Aoki et al., 2012). The increased expression level of these receptors at excitatory synapses on pyramidal neurons of the dorsal CA1 correlates significantly and negatively with the animal’s ABA vulnerability, measured as the extent of running and body weight loss (Aoki et al., 2014). Clinical studies indicate that benzodiazepines are not efficacious in treating anorexia nervosa (Steinglass et al., 2014). Unlike the α1βγ2-GABA_*A*_Rs, α4βδ-GABA_*A*_Rs are insensitive to benzodiazepines. Given the tight correlation between the gain of resilience to ABA (less running, better body weight retention) and α4 subunit expression, a drug that boosts α4βδ-GABA_*A*_R function rather than of benzodiazepines that boost α1βγ2-GABA_*A*_Rs may be more efficacious for treating anorexia nervosa.

The correlations between α4 subunit levels and wheel running and body weight loss are absent in the ventral CA1 (Aoki et al., 2018). This was a surprise, since the ventral CA1 is linked synaptically with the mPFC (Carreno et al., 2016) and through a third brain region, the thalamic nucleus reuniens (Varela et al., 2014). Excitatory afferents from ventral hippocampus to mPFC may not be the strongest contributors of pyramidal neurons’ excitability in the mPFC projecting to DMS or DR during ABA, even though this pathway is important for the antidepressant effects of ketamine (Carreno et al., 2016). Alternatively or in addition, another neurotransmitter system or another GABAR subtype may play a stronger role in modulating excitability of pyramidal neurons in the ventral hippocampus.

**FIGURE 3:**
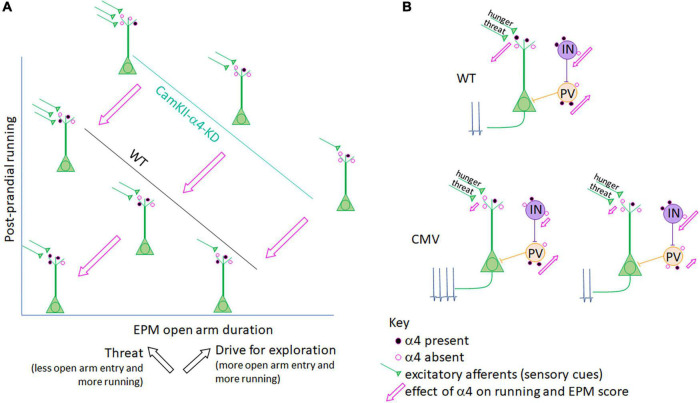
Interpretation of a α4 co-regulation of running and anxiety/exploration in ABA mice. **(A)** Axes and line plots for CaMKIIα promoter-driven a4-knockdown (KD) mice (teal line) and LoxP-negative wildtype littermates (WT, black line) are an abstraction of Supplementary Figure 1C, depicting a negative correlation for both groups between post-prandial running and the amount of time mice venture into the open arm of the elevated plus maze (EPM). While the EPM is most commonly used as a measure of anxiety-like behavior, it is important to note that hunger, while itself a stressor, promotes exploration (Atalayer and Rowland, 2011; Gutierrez, 2013; Chowdhury et al., 2021). Thus, the EPM score reflects both anxiety-like behavior (which is associated with ABA running) and exploration (also associated with ABA running; black arrows). α4-KD specifically at excitatory synapses on pyramidal cells increases both running (Supplementary Figure 1B) and open arm entry (Supplementary Figure 1C, Bar graphs), indicating that the presence of α4 (represented by the filled pink circles on WT control mouse pyramidal cells) reduces running as well as open arm entry while still preserving the correlation between the two factors (pink arrows). In both WT and CaMKIIα promoter-mediated α4-KD mice, position along the correlation line is likely determined by input from excitatory afferents (green) to pyramidal cells which convey hunger and threat signals. The Aoki lab has shown that increased α4 at excitatory synapses on hippocampal pyramidal neurons correlates negatively with running (Aoki et al., 2014, 2017) (lower left schematic, closest to where the two axes intersect), via the known mechanism of α4βδ-GABA_A_Rs to mediate shunting inhibition of pyramidal neurons (Shen et al., 2007, 2010). **(B)** When the target of α4-KD is expanded from pyramidal cells to also include GABA-IN and astrocytes, as is the case in the CMV promoter-driven α4-KD mice, the correlation between running and anxiety/exploration is lost. This may be because GABA-IN both directly inhibit pyramidal cell firing (as do PV cells, peach) as well as promote pyramidal cell firing via disinhibition (purple cell), both of which may be suppressed by α4βδ-GABA_A_Rs at excitatory synaptic inputs to GABA-IN and be de-suppressed by α4-KD in GABA-IN. Net pyramidal cell firing, depicted by action potentials, would thus depend on the ratio of transduction in GABA-IN subtypes (lower level provides two examples). Pink arrows depict the effect of α4 presence at excitatory synapses on running (y-axis) and EPM duration in the open arm (x-axis) in panel **(A)**.

α4 knockdown (α4-KD) within pyramidal neurons of dorsal CA1 (Santiago et al., 2018a) (Supplementary material methods and Figure 1) results in significant increase of running, specifically during the post-prandial hours of ABA2. These animals also increase the time spent and frequency of visiting the open arm of the elevated plus maze (EPM). Although time/frequency spent in the open arm is interpreted commonly as a reflection of the *reduction* of an animal’s anxiety, it could also be interpreted as an animal’s *increased* motivation to explore (Chowdhury et al., 2021). We believe that both the increased post-prandial running and the increased time spent in the open arm by mice with localized KD of α4 in pyramidal neurons of the hippocampus, relative to the behavior of animals with intact α4 expression (Figure 3A) are reflective of increased exploratory behavior. In support of this idea, many researchers and clinicians in the field of ABA and anorexia nervosa research consider that wheel running by laboratory animals and hyperactivity by hungry individuals reflect innate foraging-like behavior, which are exploratory (Atalayer and Rowland, 2011; Gutierrez, 2013). Adolescent female mice with global knockout of α4 subunits (α4-KO) exhibit greater ABA vulnerability, also measured as enhanced running during the first of the three days of food restriction (Chen et al., 2018a). These results support the idea that α4βδ-GABA_*A*_Rs mediate dampening of excitability of CA1 pyramidal neurons, which in turn suppresses food restriction-evoked hyperactivity. Global α4-KO may also result in greater ABA vulnerability due to the loss of dampening of the excitatory outflow elsewhere, such as amygdala, generating exacerbated anxiety, which propels wheel running (Wable et al., 2015).

EPM analysis reveals strong negative correlation between postprandial running and the duration/frequency of wildtype animals with intact α4βδ-GABA_A_Rs to enter the open arm (Supplementary Figure 1C, 3^rd^ and 4^th^ panels and Figure 3A), confirming earlier findings that wheel running is reflective of enhanced anxiety (Wable et al., 2015) and/or reduced exploratory behavior (Chowdhury et al., 2021). This correlation is equally strong among animals with α4-KD via the CaMKIIα-promoter of pyramidal neurons but is shifted to the right (Figure 3A and Supplementary Figure 1C). The rightward shift caused by α4-KD in pyramidal neurons indicates that the presence of α4βδ-GABA_A_Rs has a dual effect: reduce running, confirming earlier results (Aoki et al., 2014, 2017) but increasing anxiety, also confirming earlier results regarding hippocampal α4βδ-GABA_A_Rs of adolescent females (Shen et al., 2007).

Although α4-KD via the CaMKIIα promoter exacerbated wheel running during post-prandial periods, the same treatment reduced running during the 2 h of food availability of ABA2 (Supplementary Figure 1B). Why is the α4βδ-GABA_A_R modulation bidirectional for running? This may be associated with the enhancement of hippocampus-dependent cognition, reported previously of α4-KO animals (Moore et al., 2010; Shen et al., 2010). The decision to suppress running during the hours of food availability is acutely important for survival, requiring that animals remember the temporal aspect of food availability. The hippocampus has been shown to participate in cognition requiring motor actions that are of optimal timing: animals are hypothesized to achieve this via connectivity with DMS (Yin and Troger, 2011), the very structure where dopamine D1 receptors must be expressed for entraining circadian rhythm with hours of food availability (Gallardo et al., 2014). Moreover, DMS is a recipient zone for mPFC’s corticofugal pathway regulating food restriction-evoked hyperactivity, as was described above (Santiago et al., 2021). α4-KD from pyramidal neurons of the dorsal hippocampus may enhance this form of contextual memory revolving timing for regulating behavior. The enhanced frequency and duration in the open arm of the EPM by the group of animals with α4-KD in the pyramidal neurons of the hippocampus, relative to the wildtype group with intact α4βδ-GABA_*A*_Rs (Figure 3A and Supplementary Figure 1C, top 2 panels), may be another reflection of enhanced hippocampus-dependent cognitive behavior – namely venturing into open space to forage, when hungry. At this point, one might ask whether up-regulation of α4βδ-GABA_A_Rs, seen among ABA animals that have gained resilience, is helpful or not for survival. Our opinion is that it is helpful for gaining resilience against ABA, mainly because it reduces hyperactivity, which is linked strongly to body weight loss. Up-regulation of α4βδ-GABA_A_Rs may also reduce exploratory behavior, impair cognitive functions of the hippocampus and exacerbate anxiety-like behavior, but for animals in captivity and humans battling with anorexia nervosa, this change may be less important for survival.

In sharp contrast to the behavioral consequences observed with α4-KD in pyramidal neurons of the hippocampus, the correlation between running and elevated plus maze behavior is absent among animals with the CMV promoter-mediated α4-KD in multiple cell types (Supplementary Figure 1D). This results in α4-KD not only in pyramidal neurons but also in GABA-IN and glia. This observation suggests that α4βδ-GABA_A_R expression by non-pyramidal cells contribute significantly to the link between anxiety and wheel running, because if non-pyramidal cells did not contribute to this link, then α4-KD by the CMV-promoter would have yielded the same behavioral outcome as the α4-KD by the CaMKIIα promoter (Santiago et al., 2018a). This finding with the CMV-promoter echoes what we learned from analyzing amygdala of animals that had experienced ABA, namely that α4βδ-GABA_A_R become up-regulated in GABA-IN but not in pyramidal neurons, thereby potentially enhancing excitability of glutamatergic efferent neurons through disinhibition. The loss of correlation is likely due to the diverse influence that α4βδ-GABA_A_R have on pyramidal neurons versus parvalbumin+ GABA-IN forming strong axo-somatic inhibition upon pyramidal neurons versus GABA-IN that excite pyramidal neurons through disinhibition (Figure 3B). A study that targets α4-KD specifically in GABA-IN and glia would be needed for further elucidation of the way that the E/I balance in the hippocampus affects exploratory behavior, anxiety-regulation and wheel running.

Not only GABARs but GABAergic axon terminals forming axo-somatic and axo-dendritic inhibitory synapses onto CA1 pyramidal neurons increase following ABA induction. This morphological change is accompanied by increases in the frequency of miniature inhibitory postsynaptic currents (IPSCs) onto CA1 pyramidal neurons (Chowdhury et al., 2019). Furthermore, as was shown for the mPFC→DMS pathway, those individuals with the greatest expansion of the GABAergic axo-somatic synapses on CA1 pyramidal neurons were the most resilient, based on the minimal food restriction-evoked running and body weight loss (Chowdhury et al., 2013, 2019).

## Discussion

### Is ketamine the magic bullet to cure activity-based anorexia?

These results point to the complex relationship between the excitation-to-inhibition balance (E/I) and ABA vulnerability: boosting the GABAergic system in the mPFC and dorsal hippocampus is predicted to suppress the maladaptive behavior of exercise but also suppress food consumption. Conversely, enhancement of the excitatory outflow from the two regions is predicted to exacerbate the maladaptive running but may also enhance food consumption. How can plasticity in opposite directions within a single structure such as the mPFC be achieved? Ketamine may be the magic bullet, since a single injection of this drug to adolescent female mice (P42) evokes long-lasting (>14 days) enhancement of food consumption, which, in a long-term, increases body weight AND reduced running (Chen et al., 2018b) (Figure 2C). This echoes a small number of clinical cases reporting that ketamine ameliorates symptoms of anorexia nervosa (Mills et al., 1998; Scolnick et al., 2020; Calabrese, 2022). The mechanism of action of ketamine upon ABA and other animal models of mental illness remains to be elucidated (Zorumski et al., 2016), but initial observations indicate the involvement of drebrin A, an F-actin binding protein enriched at excitatory synapses (Aoki et al., 2005). Pharmacological blockade of NMDARs with NMDAR antagonisis elevates drebrin A, F-actin and the NR2A-containing NMDA receptors (NMDAR) at excitatory synapses (Aoki et al., 2003; Fujisawa et al., 2006) and of the concomitant decline of NR2B-containing NMDAR (Fujisawa and Aoki, 2003). Genetic deletion of drebrin A eliminates the NMDAR blockade-evoked increase of NR2A-NMDARs (Aoki et al., 2009), indicating that drebrin A is required for the activity-dependent homeostatic plasticity at excitatory synapses. Drebrin A level is augmented >14 days later at excitatory synapses of both pyramidal and GABA-IN in layer 1 of PFC in response to the single injection of an efficacious dose of ketamine in directions that could promote circuits supporting adaptive behaviors (Temizer et al., 2022).

How might ketamine achieve reduced wheel running AND increased food consumption, if this requires enhanced GABAergic inhibition of one pathway (mPFC→DMS) and reduced GABAergic inhibition of another (mPFC→DR)? Ketamine is an NMDAR open-channel blocker: it will antagonize only those NMDARs that are activated. Our working hypothesis is that ketamine resets the E/I balance in mPFC by first blocking those excitatory synapses that are hyperactive during maladaptive behaviors. The synapses that could be hyperactive and maladaptive are the excitatory synapses onto pyramidal neurons forming the mPFC→DMS pathway (causing excessive exercise) and excitatory synapses onto GABA-IN innervating pyramidal neurons forming the mPFC→DR pathway (suppressing feeding) (Figure 2C). In retrospect, the choice of injecting ketamine in the midst of FAA and within an hour of the feeding period (Chen et al., 2018b) may have been the best time to block the maladaptive behaviors of FAA and suppressed feeding. The sustained presence of drebrin A at synapses, combined with the temporally windowed blockade of synapses underlying maladaptive behaviors may enable individuals to switch mPFC’s excitatory synaptic flow and behavior from the maladaptive to an adaptive type (exercise less and eat more), both of which can be supported through plasticity of mPFC synaptic circuitry (Figure 2C).

### Recurrence of the GABAergic system as a key player of plasticity underlying the gain of activity-based anorexia resilience

Within cortical circuitry, maturation of the GABAergic system lags behind that of the excitatory neuronal system, stemming from the delay in migration of neurons to the final cortical layer (Reh et al., 2020). This delay is particularly pronounced for associational areas of cortex, such as mPFC and hippocampus (Pattwell et al., 2012; Bicks et al., 2020). This delay in maturation is likely to also delay closure of the window for experience-dependent developmental synaptic plasticity of the GABAergic system, relative to the glutamatergic system. In agreement with this idea, we observed that the gain of resilience to ABA1 in mid-adolescence correlated strongly with boosting of the GABAergic system, comprised of expansion of the GABAergic axon terminals forming axo-somatic and axo-dendritic synapses, increased GABA release probability (Chowdhury et al., 2013, 2019) and up-regulation of α4βδ-GABA_A_ receptors (Aoki et al., 2012, 2014, 2017; Chen et al., 2018a).

Stemming from this observation that some but not all animals gain resilience through plasticity of the GABAergic system, we propose that the mechanism of action of ketamine for converting the vulnerable to become resilient might also be to boost GABAergic synaptic plasticity. Among the GABA-IN in cortex, the parvalbumin+ neurons, which form axo-somatic synapses that we had analyzed in mPFC exhibit the highest spiking rates of all neurons and are exquisitely sensitive to sensory experience (Takesian and Hensch, 2013). Following the undisputed notion that ketamine is an open-channel NMDAR blocker (Luscher et al., 2020), we hypothesize that ketamine is likely to suppress excitatory synapses formed on parvalbumin^+^ GABA-IN. The notion that GABA-IN are the favored targets of sub-anesthetic doses of ketamine is supported by much evidence that are summarized in excellent reviews elsewhere (Gerhard et al., 2020; Luscher et al., 2020). The cascade of events that follow disinhibition are widely accepted to begin with the rise of extracellular glutamate, which evokes de-suppression of the mTOR protein synthesis pathway (Li et al., 2010) that yields up-regulation of synaptic proteins needed for strengthening synapses and for the *de novo* formation of excitatory and inhibitory synapses (Luscher et al., 2020). An important question that remains unanswered is how the synapses subserving adaptive behaviors are strengthened selectively over the synapses comprised of the maladaptively active synapses. Among those investigating the mechanism of action of ketamine as an antidepressant, there are competing theories regarding the cell types undergoing synapse strengthening [pyramidal versus GABA-IN; excitatory synapses versus inhibitory synapses (Luscher et al., 2020)]. We surmise that both pyramidal and GABA-INs and both excitatory and inhibitory synapses could undergo changes to support adaptive behavior (Figure 2C). For the endpoint of suppressing food restriction-evoked hyperactivity, two forms of synaptic plasticity could be evoked by ketamine: enhancement of the excitatory synapses on parvalbumin+ GABA-INs that target pyramidal neurons forming the mPFC→DMS pathway or strengthening the GABAergic inhibitory synapses on these pyramidal neurons. For the endpoint of enhancing food consumption, the one form of synaptic plasticity that is compatible with the known effects of ketamine is enhanced excitatory input to pyramidal neurons forming the mPFC→DR pathway.

For mice that have undergone ABA, the removal of the wheel from the environment and *ad libitum* food access have been sufficient for some of the animals to improve recovery and attain resilience against a second ABA-inducing environment. For humans, the “set and setting” attained through cognitive behavioral therapy that encourages mindful pursuit of adaptive behaviors is likely to be critical for successful ketamine treatment.

### Outstanding questions

This Mini-Review has introduced many speculative points of view that we believe to be worthy of future studies. As pointed out at the onset, anorexia nervosa is associated with unacceptably high rates of relapse, especially for those that have reached adulthood at the time of diagnosis (Walsh, 2013). This Mini-Review identified the GABA system as main players contributing to the gain of resilience to ABA during mid-adolescence. This Mini-Review also revealed ABA1 in late adolescence as a useful animal model for studying the heightened vulnerability and higher relapse rate observed among the adult populations with the diagnosis of anorexia nervosa. Future studies could combine the ideas that have emerged from these two observations by testing whether mechanisms that boost plasticity of the GABA system in adulthood may ameliorate the heightened relapse rate in adulthood.

How might the GABA system be boosted? BDNF (brain-derived neurotrophic factor) is recognized to boost plasticity of both GABAergic and glutamatergic synapses [reviewed in Chen et al. (2017) and Luscher et al. (2020)]. What is remarkable about ketamine is its lasting ameliorative effect on ABA (>14 days) and as an anti-depressant (>1 week), even though ketamine is cleared from the brain in less than a day. The increased release of BDNF during ketamine exposure, followed by the lasting up-regulation of BDNF is hypothesized to mediate ketamine’s immediate and sustained effects as an anti-depressant [reviewed in Luscher et al. (2020)]. Pursuit of the role of BDNF and other mechanisms (e.g., mTOR protein synthesis pathway) hypothesized to be at work for ketamine as an antidepressant should be tested within the context of ABA as well.

Unfortunately, ketamine that is administered during ABA2 in late adolescence (P56) is not as efficacious (Aoki, 2020) as when administered during ABA1 at mid-adolescence (Chen et al., 2018b). Animals experiencing ABA2 in late adolescence need multiple (instead of a single) doses separated by 24 h (Dong et al., 2022) and this treatment is still insufficient for reducing body weight loss. What might be the reason for this age-dependent change in responsiveness to ketamine? One possibility is the age-dependent decline in the expression of BDNF, even if stimulated by ketamine. Some studies indicate that ketamine binds preferentially to NR2B-containing NMDARs (Miller et al., 2014). If so, might the NR2B versus NR2A-containing NMDARs continue to undergo developmental decline into adolescence within associational cortex, as is seen during pre-adolescent ages for the primary sensory cortices (Erisir and Harris, 2003)? And if so, are glutamatergic and GABA neurons targeted differently by ketamine at their excitatory synapses? α4βδ-GABA_A_Rs have been shown to undergo surges in the expression in the hippocampus as female mice transition from juvenile to puberty stages (Shen et al., 2007). Might the PFC undergo developmental changes during adolescence as well? All or any of these changes could ultimately affect the E/I balance, among other developmental changes that make the brain less responsive to ketamine’s blockade-induced plasticity. The age-dependent efficacy of ketamine in treating ABA may generalize to studies using other animal models of mental illnesses, including depression. Our findings serve as a reminder that behavioral, synaptic and molecular analyses using animal models must be conducted across multiple time points during adolescence and into adulthood for progressing from preclinical to translational medicine.

What would be most helpful in understanding the mechanism of action of ketamine is to be able to mark those synapses and pathways that have been active during the hours that animals are expressing maladaptive behavior, and then to be able to assess whether those same synapses are targeted preferentially by ketamine. TRAP2 is a new histological approach that enables neuroscientists to label the ensemble of neurons that are active during a particular behavioral state. Those same neurons can then be transduced to express the inhibitory DREADDs (Ye et al., 2017), which, then, can be suppressed using the inhibitory DREADDs ligand during maladaptive behaviors. Our prediction is that this experimental manipulation will replicate ketamine’s effect of suppressing the synaptic circuits underlying the maladaptive ABA behavior of hyperactivity and suppressed feeding, thereby promoting healthy body weight maintenance. Our working hypothesis regarding ketamine’s mode of action could be tested by determining whether DREADD-mediated suppression of the circuit underlying the maladaptive behavior occludes ketamine’s ameliorative effects.

Further studies are needed to explore the dosing schedule that may be ameliorative during late adolescence. There are at present only three studies reporting on the efficacy of ketamine in treating anorexia nervosa. One was from more than 20 years ago, indicating that 9 out of 15 subjects with treatment-resistant anorexia nervosa showed improvements in their compulsivity depression scores (Mills et al., 1998). Another is a case study reporting a cure when ketamine was combined with ketogenic diet (Scolnick et al., 2020). This report of the case study was followed up with a pilot study of 7 subjects that underwent the combination of ketogenic diet, followed by multiple ketamine infusions: a success was seen in 4 of them (Calabrese, 2022). Whether ketamine, alone, would have been equally ameliorative and whether ketogenic diet alone might also have been efficacious are equally important questions to address. Ketogenic diet exists as one of the oldest (>100 years old) successful treatments for medically intractable epilepsy and its efficacy is hypothesized to be through boosting GABA synthesis within brain, among multiple other systems that are altered (Bough and Rho, 2007).

What, if any, are the contributions made by the ventral hippocampus in the gain of resilience against ABA? There is strong evidence to support the inter-dependence between dorsal and ventral hippocampus during spatial navigation, since inactivation of the ventral hippocampus, which is widely recognized to be involved more in anxiety-based behaviors, does impair spatial navigation (Lee et al., 2019). Future studies that probe for connections between the dorsal and ventral hippocampus as animals exhibit maladaptive versus adaptive behaviors could help with elucidating the inter-dependence of the two regions of the hippocampus during ABA induction and the gain of resilience against ABA.

What are the cellular and molecular substrates generating the diversity of ABA vulnerability during ABA1 in mid-adolescence? Might these individual differences be laid down in the subcortical regions, such as the dorsolateral and central amygdala and striatum that mature prior to puberty, with more associational cortical regions, such as the hippocampus and mPFC overlaying suppression of the maladaptive behaviors through residual synaptic plasticity that persists into adolescence? If so, how might early life experience influence synaptic functions of subcortical regions? Such questions are actively being sought (Bale et al., 2010; Fareri and Tottenham, 2016; Santiago et al., 2017, 2018b; Walker et al., 2017) and are amenable to being combined with the adolescent ABA paradigm.

## Data availability statement

The original contributions presented in this study are included in the article/Supplementary material, further inquiries can be directed to the corresponding author.

## Ethics statement

This animal study was reviewed and approved by University Animal Welfare Committee of New York University Washington Square Campus.

## Author contributions

CA and AS contributed to research design, data collection, data analyses, and consent to all aspects of the final version of the manuscript. CA wrote the first draft, which was revised by AS. Both authors contributed to the article and approved the submitted version.

## Abbreviations

ABA: activity-based anorexia
α4-KD: knockdown of the α4 subunit of GABAA receptor
α4-KO: knockout of the α4 subunit of GABAA receptor
BDNF: brain-derived neurotrophic factor
CA1: CA1 field of hippocampus
CaMKIIα: calcium-calmodulin dependent protein kinase subtype IIα
CMV: cytomegalovirus
DMS: dorsomedial striatum
DR: dorsal raphe
DREADD: designer receptors exclusively activated by designer drugs
E/I: balance excitation-to-inhibition balance
EPM: elevated plus maze
FAA: food anticipatory activity
GABA: gamma-aminobutyric acid
GABAR: GABA receptor
GABA-IN: GABA interneurons
KORD: kappa opioid receptor DREADD
PFC: prefrontal cortex
mPFC: medial prefrontal cortex
mPFC→DMS: pathway originating in medial prefrontal cortex and projecting to dorsomedial striatum
mPFC→DR: pathway originating in medial prefrontal cortex and projecting to dorsal raphe
NMDA: N-methyl-D-aspartate
NMDAR: NMDA receptor.
