# Supplementary material for: Pathway-specific GABAergic inhibition contributes to the gain of resilience against anorexia-like behavior of adolescent female mice
Source: Front Behav Neurosci. 2022 Oct 13;16:990354. doi: 10.3389/fnbeh.2022.990354 (PMC9606475; doi:10.3389/fnbeh.2022.990354)
Supplement: Supplementary file 1 [file Data_Sheet_1.docx]

**Supplemental Material**

**Materials and Methods for GABAR α4-subunit knockdown in dorsal hippocampus**

**Materials**. All mice were females. Mice were floxed mutants, possessing *loxP* sites flanking exon 3 of the Gabra4 gene, obtained from The Jackson Laboratory (JAX Stock #6874). Experimental animals were born from breeders heterozygous for the floxed mutation. Only females homozygous for the mutation were assigned to be experimental animals. Controls were WT littermates or non-littermate age-matched WT animals. Three AAVs were used: AAV2 with CaMKIIα (0.4 and 1.3) promoter driven EGFP-T2A-iCre (Cat No. VB1416 and VB1918, 1×10¹³ GC/ml, VectorLabs) to target pyramidal neurons; and AAV8/2 with CMV promoter driven eGFP-iCre (Cat No. 7097 and 7088 from VectorLabs, 1.0x10^13^ GC/ml) to target all cell types. For additional control, WT littermates were injected with pAAV-CaMKII⍺-eGFP (gift from Bryan Roth; Addgene viral prep #50469-AAV8; RRID: Addgene_50469, 1×10¹³ vg/mL).

**Methods.** α4-KD was achieved by injecting AAV-CaMKII-EGFP-iCre to target pyramidal neurons (Wang et al., 2013) (N=10, green) or AAV-CMV-EGFP-iCre to target multiple cell types, including GABA-IN and glia (van den Pol and Ghosh, 1998) (N=6, magenta) into homozygously floxed α4 mice (JAX Stock #6874) at the coordinate of AP -1.9; ML ±1.6; DV 1.7 at the age of postnatal day 26. WT littermates were injected with AAV-CMV-EGFP-iCre or (n=6) or with pAAV-CaMKII⍺-eGFP (n=2). The volume was 120 nl/hemisphere, using a Nanoject set at a flow rate of 9.2 nl/min. The procedure was performed under isoflurane anesthesia (3% for induction, 1% for maintenance), followed by IM Maloxican SR. Further details of the surgical procedure and recovery were as described elsewhere (Santiago et al., 2021). Animals underwent two rounds of ABA as described in this review (Chowdhury et al., 2013).

**Supplemental Figure 1 Caption**

ABA running, anxiety and venturing behavior are altered differently depending on the cell-type in anterio-dorsal hippocampus that were targeted for knockdown (KD) of α4 subunits of GABA_A_Rs.

(A): α4-KD was achieved by injecting AAV-CaMKII-EGFP-iCre to target pyramidal neurons (Wang et al., 2013) (N=10, green) or AAV-CMV-EGFP-iCre to target multiple cell types, including GABA-IN and glia and primarily non-pyramidal neurons (van den Pol and Ghosh, 1998) (N=6, magenta) into homozygously floxed α4 mice. The four-paneled fluorescent images show representative views of the GFP expression patterns reflecting cell type-specificity of AAV transduction. AAV-GFP-iCre was injected as controls of viral transduction in other animals (not shown).

(B) Wheel running of CaMKII-α4-KD mice (green) and CMV-α4-KD mice (magenta) was compared to that of WT littermates (black). Wheel count of 2654.86 = 1 km. Wheel running during each of the time segments is referred to as SOA (severity of ABA), quantified as an individual’s increase in wheel activity relative to baseline wheel running during the last two days of the acclimation period. The top graph shows the day-by-day increase in FAA, as animals accumulate body weight loss. Neither CaMKII- nor CMV-promoter-mediated α4-KD alter this pattern of FAA. Statistically significant differences in running were observed following pyramidal cell α4-KD subunits on the 3^rd^ day of FR of ABA2 (asterisks indicate p<0.05) for the post-prandial hours (bottom graph) and the food hours (FdHr, middle graph). Generalized α4-KD by the CMV-promoter was not effective in altering wheel running, relative to WT controls’ in all but post-prandial running on the 4^th^ day of FR of ABA2, for which there was a significant decrease in running, vs WT subjects.

(C) EPM: α4-KD in pyramidal as well as multiple cell types increased the mean frequency of mice entering the open arm. Duration in the open arm of EPM was also increased by α4-KD in pyramidal neurons and marginally also by α4-KD generally in multiple cell types. Post-prandial SOA correlates with the % frequency in the open arm of animals with α4-KD in pyramidal neurons but not of animals with α4-KD in multiple cell types. The strong negative correlation between post-prandial wheel running and % duration in the open arm of EPM is disrupted by α4-KD in multiple cell types but not in animals with α4-KD in pyramidal neurons, suggesting a role for non-pyramidal α4 expression in determining individual differences in anxiety. * indicates p<0.05; # indicates 0.05<p<0.1. ** indicates p<0.01; **** indicates p<0.0001.
