# Supplementary figures and images for: Pathway-specific GABAergic inhibition contributes to the gain of resilience against anorexia-like behavior of adolescent female mice
Source: Front Behav Neurosci. 2022 Oct 13;16:990354. doi: 10.3389/fnbeh.2022.990354 (PMC9606475; doi:10.3389/fnbeh.2022.990354)

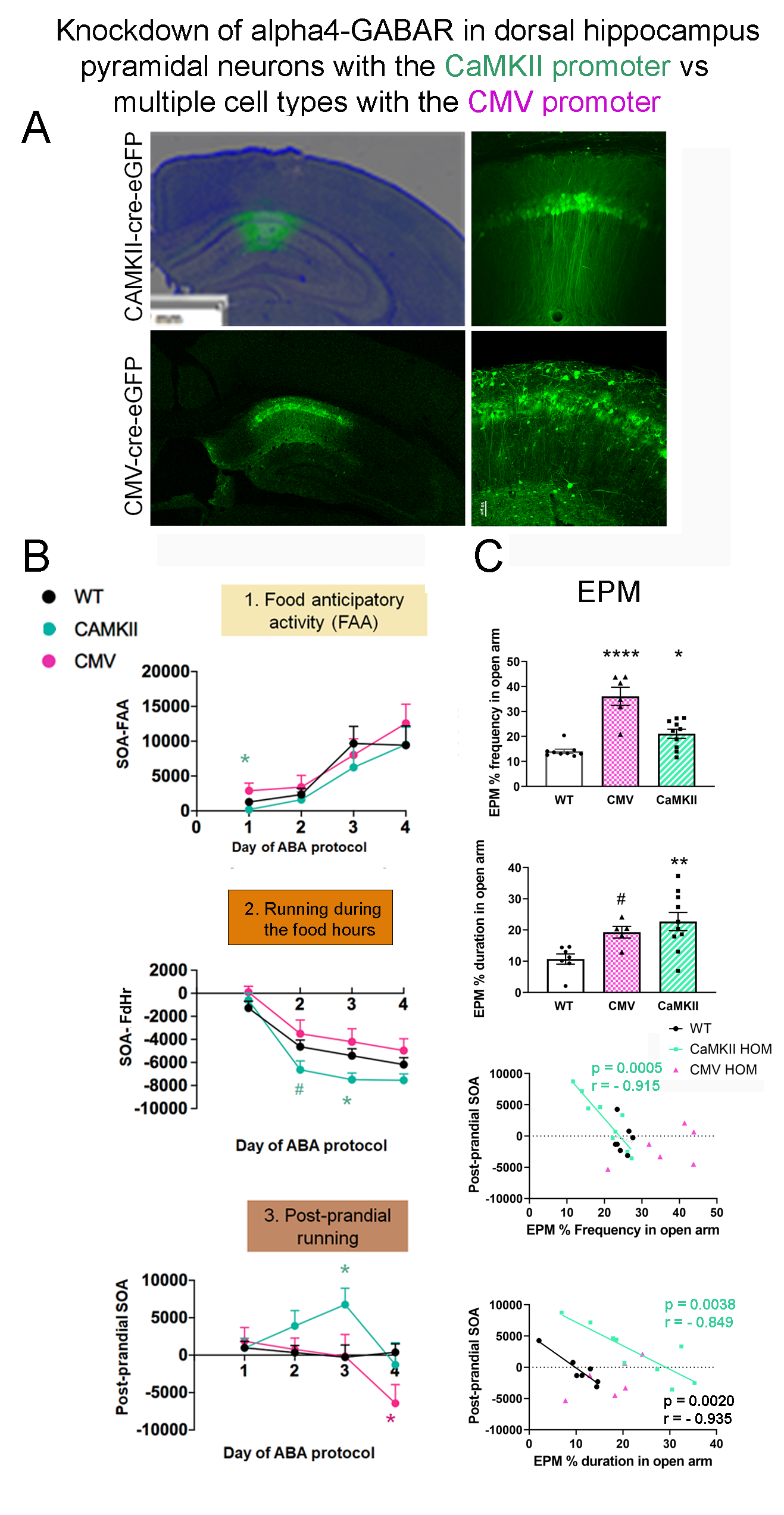

Supplement: Supplementary file 2 [file Image_1.TIF]
